# Transpedicular injection of rhBMP-2 with β-tricalcium phosphate to reduce the proximal junctional kyphosis after adult spinal deformity correction: preliminary study

**DOI:** 10.1038/s41598-024-57371-w

**Published:** 2024-03-20

**Authors:** Ohsang Kwon, Jun-Young Choi, Jin-Ho Park, Dae-Woong Ham, Sang-Min Park, Jin S. Yeom, Ho-Joong Kim

**Affiliations:** 1 Spine Center and Department of Orthopedic Surgery, Seoul National University College of Medicine and Seoul National University Bundang Hospital, 166 Gumiro, Bundang-gu, Sungnam, 463-707 Republic of Korea; 2 Department of Orthopedic Surgery, Dongguk University Ilsan Hospital, Goyang, Gyeonggido Republic of Korea; 3 Department of Orthopedic Surgery, Chung-Ang University College of Medicine, Chung-Ang University Hospital, Seoul, Republic of Korea

**Keywords:** Biophysics, Medical research, Risk factors

## Abstract

The aim of this preliminary study was to assess the impact of injecting recombinant human bone morphogenetic protein-2 (rhBMP-2) with β-tricalcium phosphate (β-TCP) carrier into the uppermost instrumented vertebra (UIV) during surgery to prevent the development of proximal junctional kyphosis (PJK) and proximal junctional failure (PJF). The 25 patients from study group had received 0.5 mg rhBMP-2 mixed with 1.5 g β-TCP paste injection into the UIV during surgery. The control group consisted of 75 patients who underwent surgery immediately before the start of the study. The incidences of PJK and PJF were analyzed as primary outcomes. Spinopelvic parameters and patient-reported outcomes were analyzed as secondary outcomes. Hounsfield unit (HU) measurements were performed to confirm the effect of rhBMP-2 with β-TCP on bone formation at preoperative and postoperative at computed tomography. PJK and PJF was more occurred in control group than study group (p = 0.02, 0.29, respectively). The HU of the UIV significantly increased 6 months after surgery. And the increment at the UIV was also significantly greater than that at the UIV-1 6 months after surgery. Injection of rhBMP-2 with β-TCP into the UIV reduced PJK and PJF rates 6 months after surgery with new bone formation.

## Introduction

Adult spinal deformity (ASD) is a major topic of interest for spinal surgeons worldwide^1^. Vast efforts are being made to understand the pathophysiology of the disease, subcategorize the deformity, set the ideal correction target, and minimize postoperative complications^2,3^. However, despite various approaches to prevent proximal junctional kyphosis (PJK) and proximal junctional failure (PJF), their incidence has not diminished significantly^4,5^.

Cement-augmented screw insertion and laminar hook techniques are believed to prevent implant or bone-instrument interface failure^4,6^. Ligamentous augmentation and careful selection of the uppermost instrumented vertebra (UIV) have been introduced to prevent ligamentous failure^7^. However, the most troublesome and frequent cause of PJF is bony failure arising from osteoporotic bone and increased stress load at the proximal junction^4^. The quality of trabecular bone at the UIV is the most crucial factor for this type of PJF. The management of underlying osteoporosis and perioperative administration of teriparatide to increase bone quality have been attempted in the field^8–10^.

Recombinant human bone morphogenetic protein-2 (rhBMP-2) has proven its efficacy in spinal fusion surgeries^11^, and bone formation in dentistry when rhBMP-2 was injected with a β-tricalcium phosphate (β-TCP) carrier was recently reported^12^. Therefore, we hypothesized that intraoperative local administration of rhBMP-2 carried by β-TCP would have a beneficial effect on bone quality around the UIV level screw and consequently reduce the incidence of bony failure-type PJF.

## Methods

### Study design and patients

This retrospective review was approved and the requirement of informed consent for rhBMP-2 usage was waived by the Institutional Review Board of Seoul National University Bundang Hospital (B-2208–777-108). Our study was performed in accordance with relevant guidelines for all participant patients. A total of 25 consecutive patients undergoing ASD reconstruction surgery between August 2021 and January 2022 were included in the study group. The control group included patients who underwent ASD correction surgery immediately before the study group between January 2020 and August 2021. The surgical indications of our group were as follows: (1) age > 50 years; (2) diagnosis of ASD with sagittal imbalance defined as sagittal vertical axis (SVA) > 5 cm, pelvic tilt (PT) > 20°, or pelvic incidence minus lumbar lordosis (PI-LL) mismatch > 20° on lateral radiographs in the standing position; and (3) subjective disability due to stooping posture. The exclusion criteria were as follows: (1) presence of other spinal diseases impeding walking, such as thoracic and/or cervical myelopathy; (2) peripheral vascular disease; (3) any syndromic or neuromuscular disease; and (4) any serious uncontrolled medical comorbidity, such as sepsis or malignancy, which would cause disability or worsen the general medical condition.

### Surgical procedures

All surgeries were performed by a single experienced surgeon using individualized yet similar surgical methods. There were no major changes in the surgical procedures or methods for patients enrolled in the study. Patients were prone positioned on a Mizuho OSI modular table system (Mizuho OSI, Union City, CA, USA) for maximal LL, and lateral radiographs were obtained. The PI-LL mismatch was calculated using this image to determine the desired correction angle. Surgical strategies, including the type of osteotomy (i.e., 3-column osteotomy or multilevel posterior column osteotomy) and fusion length, were set based on the PI-LL mismatch. Intramuscular dissection, rather than periosteal dissection, was performed at the UIV-2 level and above, to preserve the posterior ligamentous complex near the proximal junction. The UIV is usually T10, with minor variations, and sacral fusion and iliac screw insertion are almost routinely performed to sustain the long construct and avoid hastened degeneration of the L5/S1 disc. Dual rods were applied with a domino connector for stable fixation of the long construct and to prevent rod fracture. All pedicle screws used in the surgery were polyaxial type and ranged in size from 5.0 mm to 7.5 mm depending on the patient's pedicle size. The dual rod used a 6.0 mm size product made of titanium alloy and a cobalt chrome material.

### Intraoperative local administration of rhBMP-2 into the UIV

**Figure 1:**
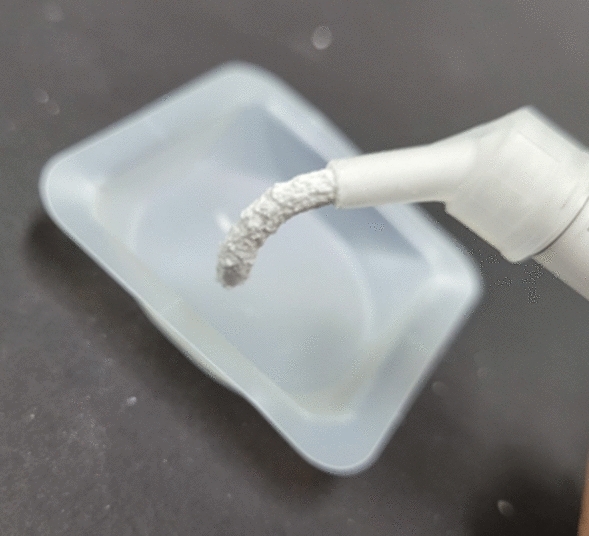
*β*-tricalcium phosphate which is used as carrier for rhBMP-2 at this study.

Pedicle screw diameter and length were anticipated using preoperative computed tomography (CT). Screws with a diameter and length 2 and 5 mm smaller, respectively, than the largest possible screw from the CT scan, were inserted at the UIV level. For example, if the CT scan showed that a 6.5 mm × 45 mm screw could be inserted into the UIV, a 4.5 mm × 40 mm screw was inserted. After screw placement at all vertebral levels was completed, an intraoperative CT scan with O-arm® (Medtronic, Minneapolis, MN, USA) was performed. Screw trajectory was then confirmed from the O-arm® image, and the screws at the UIV level were removed to make the pre-pathway of rhBMP-2 to vertebral body. Bone wax was applied at the entry hole of the previously inserted screws to block blood oozing from the trabecular bone, followed by rhBMP-2 injection. A mixture of 0.5 mg rhBMP-2 and 1.5 g β-TCP carrier (CGbio, Seoul, Korea) (Fig. 1) with 0.2 ml normal saline was carefully injected through the bone wax and into the screw hole, immediately followed by insertion of pedicle screws of definitive diameter and length. Total amount of injection was less than 0.5 cc at respective one pedicle screw hole. There was no visible leakage outside of bone wax.

### Radiographic analysis of PJK/F and sign of new bone formation

**Figure 2:**
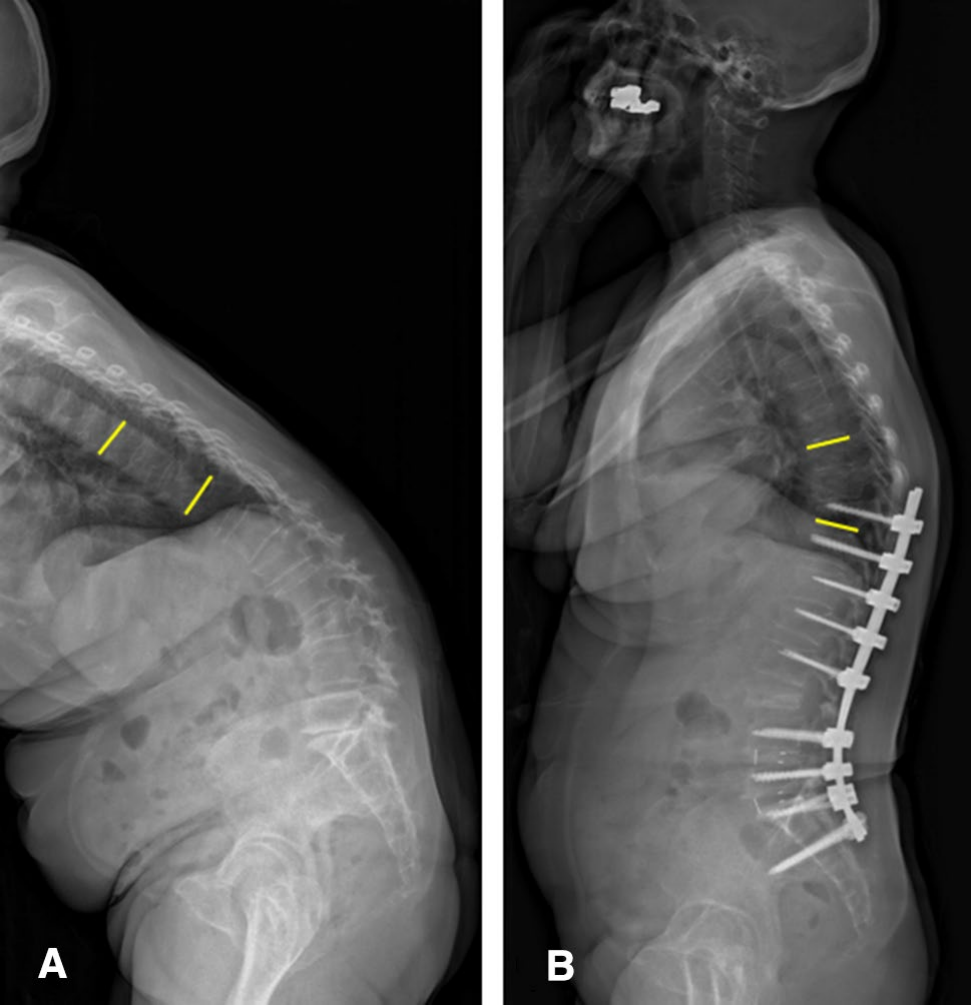
(**a**) Preoperative proximal junction sagittal Cobb angle. (**b**) Postoperative proximal junctional sagittal Cobb angle. It can be defined proximal junctional kyphosis (PJK) because it satisfies all of the following PJK definitions: (**b**) > 15° and (**b**) – (**a**) > 15°.

Spinopelvic parameters including pelvic incidence (PI), LL, PI-LL, SVA, thoracic kyphosis (TK), and the amounts of surgical correction were measured using biplanar stereo radiographic full-body imaging (EOS, Paris, France)^13^. PJK was defined as (1) postoperative proximal junctional sagittal Cobb angle > 15° and (2) change in the proximal junctional sagittal Cobb angle from the preoperative measurement of > 15°. The proximal junction is between the upper endplate of the vertebra two-level superjacent to the UIV and the lower endplate of the UIV (Fig. 2). PJK is subdivided into one of the following: (1) disruption of the posterior osseo-ligamentous complex (ligamentous failure), (2) UIV or UIV + 1 fracture (bony failure), or (3) pull-out of instrumentation (bone-implant interface failure)^14^. PJF was defined as any pain, neurological deficit, compression fracture, or implant failure necessitating revision surgery.

### Hounsfield units (HU) measurement

**Figure 3:**
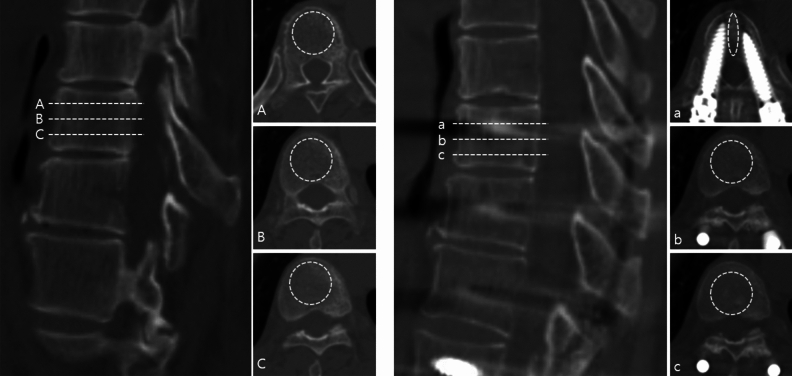
Measurement of Hounsfield Units (HU) from computed tomography using the Scheriber measurement methods. HU was measured within a circular range in the axial view in three sections divided by height. The HU of the vertebral body was defined as the average HU value of the three sections. In the vertebral body where the pedicle screw was inserted, HU was measured in an oval shape in the space between the pedicle screws. Afterwards, the process is the same.

**Figure 4:**
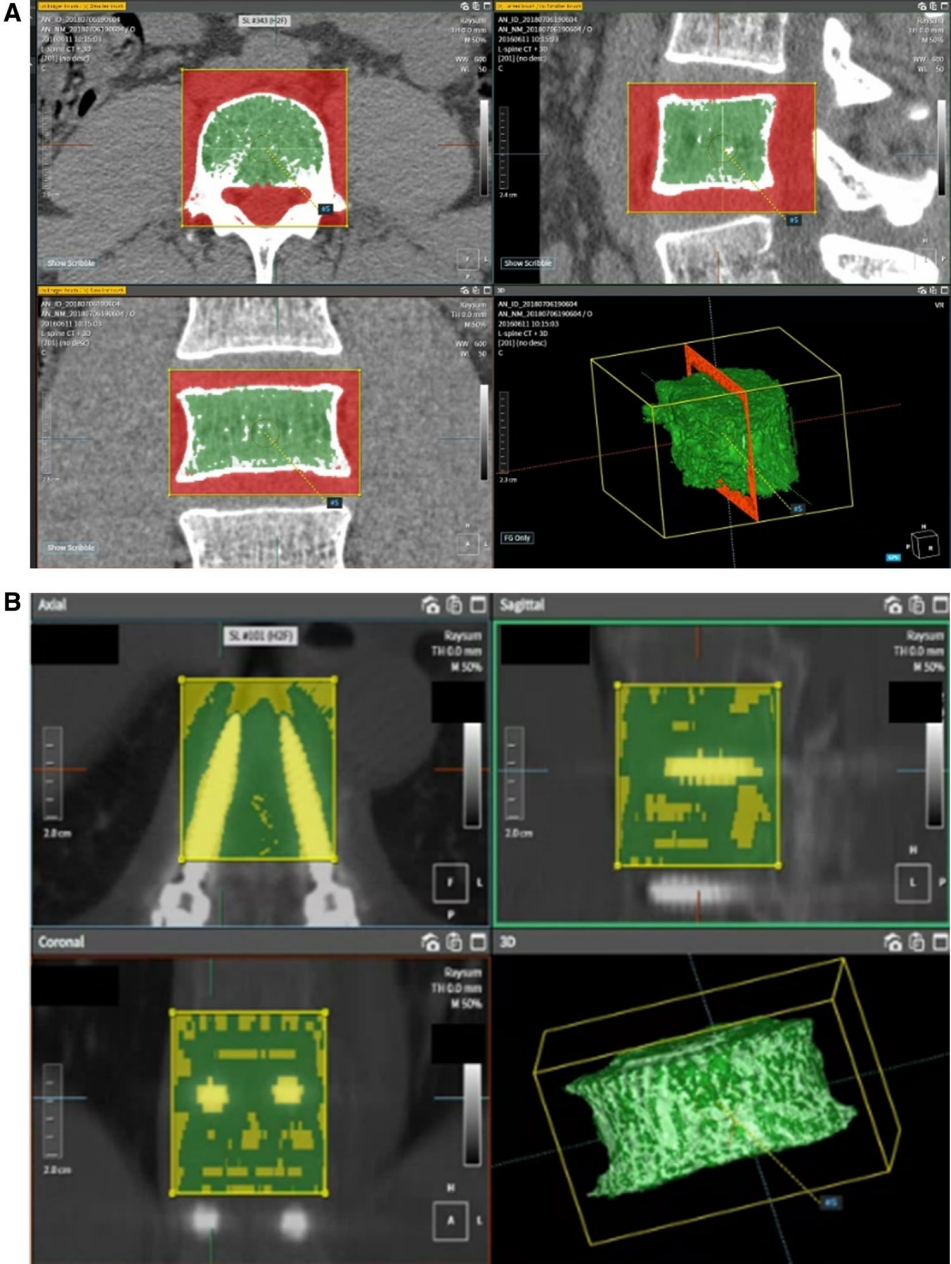
(**a**) Hounsfield Units (HU) measurement of vertebral body without pedicle screw from computed tomography using the AVIEW software. An upper limit of 1500 HU was used to exclude metal artifacts caused by screws and cortical bone, and a lower limit of 100 HU was used to exclude disc and other soft tissues. (**b**) Vertebral body with pedicle screw HU measurement. Upper and lower limit of HU get rid of pedicle screw and metal artifacts.

HU were measured from the preoperative and six month-postoperative CT scans at the UIV and UIV-1 in an integrative manner using the Picture Archiving and Communication System (PACs, INFINITT M6, Seoul, Korea) and the Coreline Aview software (v1.1.40, Seoul, Korea) in order to assess the amount of bone formation as a result of rhBMP-2 radiographically^15–17^. After obtaining informed consent about CT examination with radiation risk, follow-up CT at 6 months was performed. HU measurement using PACs is conducted according to the method outlined in Schreiber’s study. (Fig. 3)^18^. After the CT sagittal view was divided into three sections based on the vertebral body height, HU was calculated by identifying an oval-shaped ROI in the corresponding axial cut that contains only the trabecular bone. The HU of the related level vertebral body was determined using the average value of HU measured in each of the three sections. The procedure of measuring HU using Coreline Aview software was as follows (Fig. 4a and 4b): The region of interest (ROI) was set roughly by manual at the axial, sagittal, and coronal regions. Then the upper and lower limits of HU were set so that only trabecular bone in the vertebral body were included. Then, an upper limit of 1,500 HU and a lower limit of 100 HU were applied using the software to exclude the lung parenchyma, cortical bone of the vertebral body, pedicle screws, and metal artifacts. Finally, additional manual manipulation and ROI confirmation were performed to avoid measurement bias. Then, the software three-dimensionally integrated the HU of the trabecular bones of the UIV or UIV-1.

### Patient reported outcome measurements (PROMs)

The Oswestry disability index (ODI), EuroQOL (EQ-5D), and Scoliosis Research Society questionnaire (SRS-22) were used to assess surgical outcomes^19,20^. The ODI is a self-administered questionnaire that measures “back-specific function” on a 10-item scale with six response categories each^20^. The EQ-5D is a 5-dimensional health state classification; the five dimensions are mobility, self-care, usual activities, pain/discomfort, and anxiety/depression^19,21^. EQ-5D “health status was defined by selecting one level from each dimension. The EQ-5D preference-based measure can be regarded as a continuous outcome scored on a 0 to 1.00 scale, with 1.00 indicating “full health” and 0 representing death. These data were collected preoperatively and reassessed three and six months after surgery.

### Statistical Analysis

Continuous variables between the groups were compared using an independent t-test. Values are presented as mean ± standard deviation. Categorical variables were compared using the χ^2^ test. Given the mean difference in the PJK incidence ratio between the study group and the control group, a post hoc power analysis was performed with an alpha value of 0.05 using G*power 3.1^22^. All statistical analyses were performed using SPSS version 26 software (SPSS Inc., Chicago, IL, USA). Statistical significance was set at p < 0.05.

## Results

### Patient characteristics

A control group of 75 patients were included at this study. Other demographic data, including height, weight, body mass index, bone mineral density, hand grip strength, past medical histories, and osteoporosis medication, did not differ significantly between the study and control groups (Table 1).

**Table 1:** Patient characteristics, baseline radiographic and clinical parameters of the study population.

	UIV rhBMP-2	Control	Total	p-value
Number	25	75	100	
Age	73.7 ± 6.3	72.9 ± 6.4	73.1 ± 6.4	0.80
Sex, female (%)	22 (88%)	66 (88%)		1.00
Height (cm)	169.7 ± 6.2	150.8 ± 9.3	150.5 ± 8.7	0.57
Weight (kg)	55.0 ± 7.9	57.9 ± 8.8	57.2 ± 8.6	0.14
BMI (kg/m^2^)	24.6 ± 3.5	25.4 ± 2.7	25.2 ± 2.9	0.51
BMD (g/cm^2^)	0.79 ± 0.1	0.75 ± 0.1	0.76 ± 0.1	0.12
HGS (kg)	15.5 ± 5.7	16.9 ± 8.2	16.6 ± 7.6	0.39
Hypertension (%)	14 (56%)	33 (44%)	47	0.30
Diabetes melltius (%)	6 (24%)	19 (25%)	25	0.80
Cerebrovascular disease (%)	3 (12%)	10 (13%)	13	0.87
Renal disease (%)	3 (12%)	5 (7%)	8	0.40
Smoking (%)	1 (4%)	2 (3%)	3	0.74
Osteoporosis medication				
Preoperative (%)	8 (32%)	30 (40%)	38	0.48
Postoperative (%)	7 (28%)	23 (31%)	30	0.46
Spinopelvic parameters				
PI (°)	52.1 ± 11.3	53.6 ± 11.7	53.2 ± 11.6	0.58
LL (°)	-5.3 ± 24.1	7.1 ± 20.0	4.0 ± 21.7	**0.01**
PI–LL (°)	57.4 ± 26.5	47.1 ± 23.0	49.7 ± 24.2	0.07
SVA (°)	210 ± 79	182 ± 70	189 ± 73	0.10
TK (°)	13.3 ± 13.4	15.6 ± 6.8	14.8 ± 25.6	0.50
Patient reported outcomes				
ODI	22.8 ± 8.6	25.2 ± 7.4	24.6 ± 7.7	0.20
EQ-5D	0.31 ± 0.3	0.21 ± 0.2	0.23 ± 0.3	0.10
SRS-22	2.5 ± 0.6	2.2 ± 0.5	2.3 ± 0.5	0.05

UIV, uppermost instrumented vertebra; rhBMP-2, human recombinant bone morphogenetic protein 2; BMI, body mass index; BMD, bone mineral density of femur total; HGS, hand grip strength; PI, pelvic incidence; LL, lumbar lordosis; SVA, sagittal vertical axis; TK, thoracic kyphosis; ODI, Oswestry disability index; EQ-5D, EuroQOL-5 dimension; SRS-22, Scoliosis Research Society questionnaire.

p-values < 0.05 are shown in bold.

### Radiographic parameters and PJK/PJF

Preoperative spinopelvic parameters were not significantly different between the two groups except for lumbar lordosis [-5.3 ± 24.1 vs. 7.1 ± 20.0 for the study and control groups, respectively (p = 0.01)], implying smaller mean lumbar lordosis in the study group (Table 1). The PI-LL mismatch and SVA values were comparable between the two groups during the postoperative phase (Table 2).

**Table 2:** Postoperative radiographic parameters and proximal junctional kyphosis (PJF) and failure (PJF) rates.

	UIV rhBMP-2	Control	p-value
Postop spinopelvic parameters			
PI (°)	51.2 ± 9.7	51.2 ± 11.4	0.99
LL (°)	35.6 ± 9.2	33.3 ± 9.1	0.29
PI–LL (°)	15.6 ± 11.2	17.8 ± 11.1	0.39
SVA (°)	52.0 ± 31.7	67.2 ± 49.9	0.17
TK (°)	26.1 ± 5.7	25.4 ± 8.8	0.65
PJK (cases)	2 (8%)	24 (32%)	**0.02**
Ligamentous	0	0	
Bone	2	22	
Interface	0	2	
PJF (cases)	1 (4%)	11 (15%)	0.29

UIV, uppermost instrumented vertebra; rhBMP-2, human recombinant bone morphogenetic protein 2; PI, pelvic incidence; LL, lumbar lordosis; SVA, sagittal vertical axis; TK, thoracic kyphosis; PJK, proximal junctional kyphosis; PJF, proximal junctional failure.

p-values < 0.05 are shown in bold.

The incidence of PJK was 2 of 25 (8%) patients in the study group, which was significantly lower than that in the control group, that is, 24 of 75 (32%) patients (p = 0.02). The odds ratio of PJK in the study group was 0.185 (0.04–0.848, 95% CI) (Table 2). One of the two PJK cases in the study group developed bony failure-type PJF, whereas 10 of the 11 PJF patients in the control group (p = 0.29) was bony failure-type. The post hot power analysis confirmed the difference in mean and standard deviation in the PJK incidence ratio between the both groups with an alpha value of 0.05 and a statistical power of 80.0%.

### Patient reported outcome measurements

PROMs at six months postoperatively were compared between the two groups (Table 3). Patients in the study group showed better clinical outcomes, having lower ODI scores (16.7 ± 7.4 vs. 21.1 ± 8.9, p = 0.04), and higher EQ-5D (0.57 ± 0.2 vs. 0.37 ± 0.3, p < 0.01) and SRS-22 scores (3.36 ± 0.7 vs. 2.83 ± 0.7, p < 0.01).

**Table 3:** Patient reported outcome measures at six months after surgery.

	UIV rhBMP-2	Control	p-value
ODI	16.2 ± 7.4	21.1 ± 8.9	**0.04**
EQ-5D	0.57 ± 0.2	0.37 ± 0.3	** < 0.01**
SRS-22	3.4 ± 0.7	2.9 ± 0.7	** < 0.01**

UIV, uppermost instrumented vertebra; rhBMP-2, human recombinant bone morphogenetic protein 2; ODI, Oswestry disability index; EQ-5D, EuroQOL 5-dimension; SRS-22, Scoliosis Research Society-22 questionnaire.

p-values < 0.05 are shown in bold.

### Hounsfield unit measurements

**Figure 5:**
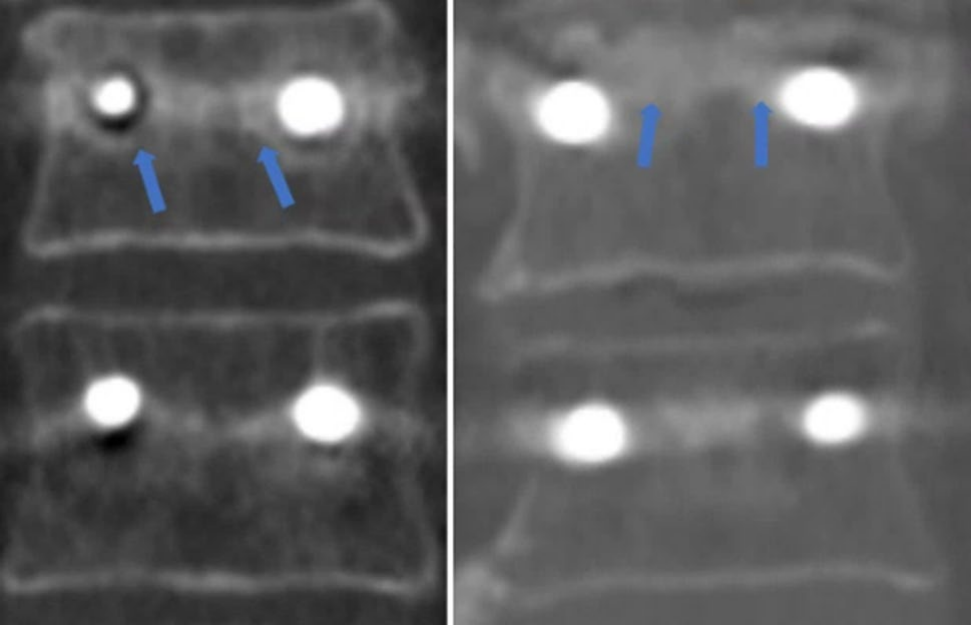
Coronal images of three representative patients of the uppermost instrumented vertebra (UIV) rhBMP-2 injection group six month after surgery. Signs of trabecular bone growth are seen around the UIV pedicle screws (arrows).

**Figure 6:**
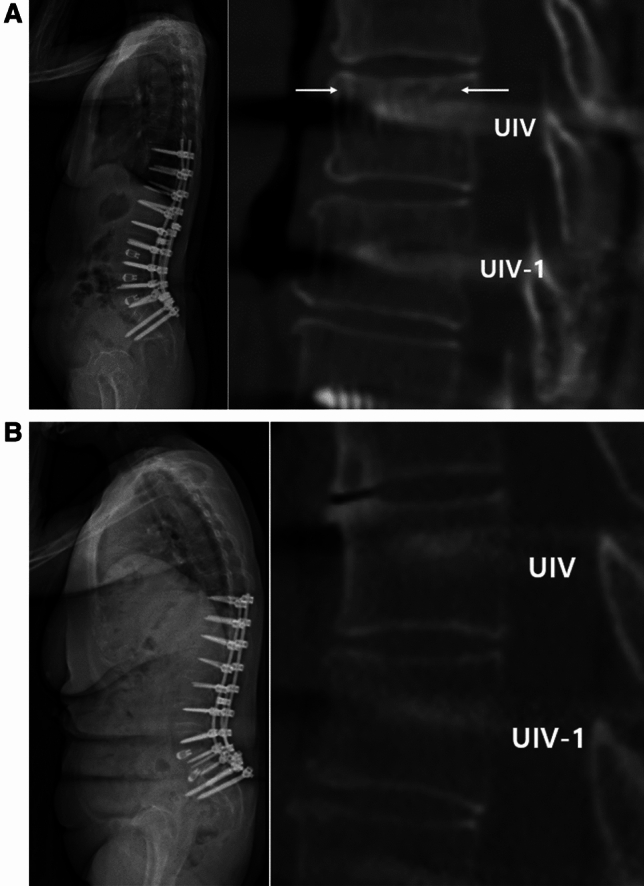
(**a**) Representative cases, patient who applied rhBMP-2 with *β*-TCP carrier showed high density at sagittal cut (arrows) of computed tomography at 6 months after surgery. (**b**) On the other hand, patient who underwent surgery without rhBMP-2 in the control group showed no enhancement around the pedicle screw at uppermost instrumented vertebra (UIV) level.

The HU was measured to radiologically confirm new bone formation by the effect of rhBMP-2. While the HU of the UIV at six months after surgery increased significantly compared with that in the preoperative scan (387.2 ± 41.9 vs. 318.8 ± 44.3, p < 0.001), HU at UIV-1 showed no significant difference (346.4 ± 45.2 vs. 330.8 ± 48.0, p = 0.250). This resulted in a significantly higher HU at the UIV compared with the UIV-1 in the 6 months' postoperative scan (387.2 ± 41.9 vs. 346.4 ± 45.2, p = 0.003). Similar findings were observed with the Schreiber’s HU measurement method. Six months after surgery, there was a statistically significant increase in HU at the UIV level compared to preoperative state. (158.8 ± 36.6 vs. 138.1 ± 40.2, p = 0.001), and there was also a difference in HU between UIV and UIV-1 in the 6 months' postoperative scan (158.8 ± 36.6 vs. 132.7 ± 26.6, p < 0.001). (Table 4). A representative cut of the postoperative CT scan for new bone formation surrounding the UIV pedicle screw where the rhBMP-2 was applied is shown in Fig. 5 and Fig. 6a. This finding is more evident when compared with the control group (Fig. 6b).

**Table 4:** Hounsfield unit measurement at the UIV and UIV-1 from the preoperative CT scans and six months postoperative CT scans of the study group.

	Preop	Postop 6 M	p-value
By using the Coreline Aview Program			
UIV	318.8 ± 44.3	387.2 ± 41.9	** < 0.001**
UIV-1	330.8 ± 48.0	346.4 ± 45.2	0.250
p-value	0.36	**0.003**	
By using the Schreiber methods			
UIV	138.1 ± 40.2	158.8 ± 36.6	**0.001**
UIV-1	134.5 ± 37.9	132.7 ± 26.6	0.777
p-value	0.323	** < 0.001**	

UIV, uppermost instrumented vertebra; CT, computed tomography.

p-values < 0.05 are shown in bold.

### Complications

There were no revision cases among the 25 patients in the study group. However, revision surgery which extended posterior fixation and fusion at least 3 upper levels and posterior decompression at PJF level were performed for neurologic deficit with PJK that occurred within 6 months after surgery in 5 of the 75 control group patients. Any subchondral sclerosis was not noticed in the study group.

## Discussion

This study presents a novel surgical strategy for preventing PJK and PJF after ASD surgery. Transpedicular injection of rhBMP-2 with a β-TCP carrier into the UIV appears to reduce the incidence of PJK by reinforcing trabecular bone formation around the pedicle screws. The enhanced bone quality of the UIV leads to higher resistance to compression fractures and stronger pullout strength of the pedicle screws. The marked decrease in the incidence of PJK led to a low occurrence of PJF during the first six months after surgery. The superior PROMs of the study group may have been rooted in the lower PJF rate since other radiographic parameters such as the amount of surgical correction and radiological outcomes were similar between the both groups.

Notably, rhBMP-2 promotes osteoinduction and enhances allograft incorporation, increasing the rate of interbody fusion^11^. More recently, the topical placement of rhBMP-2 with a scaffold resulted in bone growth in the field of oral and maxillofacial surgery^12,23^. In an animal study, injection of rhbmp-2 and bone cement into the femoral condyle resulted in bone formation through osteogenesis^24^. Localized bone augmentation through local administration of rhBMP-2 in our study group would likely share a biological mechanism. This strategy is distinct from other PJK prevention strategies, such as cement or ligament augmentation, in that it does not violate natural biology or necessitates additional anatomical exposure. Reducing the rate of PJF and, consequently, the rate of revision surgery in the ASD population would reduce the burden on patients and benefit the healthcare community by reducing the required costs of the process.

New bone formation surrounding the pedicle screw as the result of applying rhBMP-2 can also be used for other surgeries where robust integration between implant and bone is necessary. Many surgeons tried to make a stronger screw fixation strength at osteoporotic vertebra with large diameter and longer pedicle screw, cortical bone trajectory, more converge angle, with cementation. The technique of this study is thought to be one way to increase the integration strength between implant and bone^25–28^.

The majority of PJK cases occur early in the postoperative course, reported as 66% within 3 months^14^. Therefore, the fate of the proximal junction of the long-fusion construct was mostly determined during this early period. Meanwhile, the action of rhBMP-2 tracked by HU measurement in the interbody fusion setting showed that bone integration was significantly activated as early as six months postoperatively and reached a plateau at 12 months postoperatively^11^. Hence, patients in the study group were followed up six months after surgery with a CT scan to survey the incidence of PJK and search for any sign of enhanced trabecular bone formation at the UIV. For the quantitative analysis, we measured HU. Measurement of HU in the setting of vertebral body-retaining implants unavoidably encounters the risk of overmeasurement due to metal artifacts. It also depends largely on how the researcher sets the ROI*.* To overcome this issue, we utilized two different methods. All pedicle screws inserted into the patient's vertebral body were the same product, and the function of the software program was used to minimize metal artifacts. HU, which exceeds the HU of cancellous bone, was set to be automatically excluded. In this study, we set the high cut-off value as 1500 HU to minimize the intervention by metal artifacts. The significant increase in HU at UIV, even compared with UIV-1, may be a sign of new bone formation due to rhBMP-2 injection.

This study had some limitations. First, the sample size was small, and the follow-up period was short. Only 25 patients were injected with rhBMP-2 in the UIV. There were difficulties in deriving the sample size due to the lack of prior research. However, post hoc power analysis confirmed the difference in mean and standard deviation in the PJK incidence ratio between the both groups with an alpha value of 0.05 and a statistical power of 80.0%. Further studies with a larger sample size or randomized controlled trials are necessary to reach a solid conclusion. A longer follow-up would also be beneficial, although it is generally accepted that most PJKs, especially bony failure, occur less than six months after surgery. Second, the ideal dose of rhBMP-2 for this study has not been determined, as this is the first trial focused on the prevention of PJK. After conducting a comprehensive literature review, we have chosen to use 0.5 mg of rhBMP-2 in this preliminary study. This decision is based on the assumption that the required amount of rhBMP-2 may be comparable to or less than that used in lumbar fusion surgery at a single level. Third, β-TCP is a radiopaque material that may remain unabsorbed in postoperative CT scans six months after surgery. However, a recent study utilizing the same combination of rhBMP-2 and β-TCP for alveolar bone augmentation demonstrated increased radiolucency in the radiograph at three months after implantation as compared to the immediate postoperative radiograph^29^. Hence, we do not believe that β-TCP can lead to overestimation when measuring HU.

In conclusion, the intraoperative transpedicular injection of rhBMP-2 with β-TCP carrier into the UIV reduced the incidence of bony failure in the proximal junction, thus necessitating revision surgery. It is easily applicable because the technique does not require a change in the surgical strategy of the surgeon, nor does it require any significant additional surgical procedures.

### Supplementary Information

Supplementary Information.

## Data Availability

All data generated or analyzed during this study are included in this published article and its supplementary information files.
